# A Data-Driven Approach to Sugarcane Breeding Programs with Agronomic Characteristics and Amino Acid Constituent Profiling

**DOI:** 10.3390/metabo14040243

**Published:** 2024-04-21

**Authors:** Chiaki Ishikawa, Yasuhiro Date, Makoto Umeda, Yusuke Tarumoto, Megumi Okubo, Yasujiro Morimitsu, Yasuaki Tamura, Yoichi Nishiba, Hiroshi Ono

**Affiliations:** 1 Institute of Food Research, National Agriculture and Food Research Organization, 2-1-12 Kannondai, Tsukuba 305-8642, Ibaraki, Japan; 2 Graduate School of Humanities and Sciences, Ochanomizu University, 2-1-1 Otsuka, Bunkyo-ku, Tokyo 112-8610, Japan; 3 Research Center for Advanced Analysis, National Agriculture and Food Research Organization, 2-1-12 Kannondai, Tsukuba 305-8642, Ibaraki, Japan; datey262@affrc.go.jp (Y.D.); ono@affrc.go.jp (H.O.); 4 Kyushu-Okinawa Agricultural Research Center, National Agriculture and Food Research Organization, Annou 1742-1, Nishinoomote, Kagoshima 891-3102, Japan; umedam461@affrc.go.jp (M.U.); yusuke@affrc.go.jp (Y.T.); okubom014@affrc.go.jp (M.O.); 5 Institute for Human Life Science, Ochanomizu University, 2-1-1 Otsuka, Bunkyo-ku, Tokyo 112-8610, Japan; morimitsu.yasujiro@ocha.ac.jp; 6 Western Region Agricultural Research Center (Kinki, Chugoku and Shikoku Regions), National Agriculture and Food Research Organization, 6-12-1 Nishifukatsu-cho, Fukuyama, Hiroshima 721-8514, Japan; tamtam@naro.affrc.go.jp; 7 Kyushu-Okinawa Agricultural Research Center, National Agriculture and Food Research Organization, 2421 Suya, Koshi, Kumamoto 861-1192, Japan; nishibay102@affrc.go.jp

**Keywords:** sugarcane juice, amino acids, agronomic characteristics, breeding

## Abstract

Sugarcane (*Saccharum* spp. hybrids) and its processed products have supported local industries such as those in the Nansei Islands, Japan. To improve the sugarcane quality and productivity, breeders select better clones by evaluating agronomic characteristics, such as commercially recoverable sugar and cane yield. However, other constituents in sugarcane remain largely unutilized in sugarcane breeding programs. This study aims to establish a data-driven approach to analyze agronomic characteristics from breeding programs. This approach also determines a correlation between agronomic characteristics and free amino acid composition to make breeding programs more efficient. Sugarcane was sampled in clones in the later stage of breeding selection and cultivars from experimental fields on Tanegashima Island. Principal component analysis and hierarchical cluster analysis using agronomic characteristics revealed the diversity and variability of each sample, and the data-driven approach classified cultivars and clones into three groups based on yield type. A comparison of free amino acid constituents between these groups revealed significant differences in amino acids such as asparagine and glutamine. This approach dealing with a large volume of data on agronomic characteristics will be useful for assessing the characteristics of potential clones under selection and accelerating breeding programs.

## 1. Introduction

Sugarcane (*Saccharum* spp. hybrids, Poaceae) is cultivated in tropical and subtropical areas. In Japan, sugarcane is mainly produced in the Nansei Islands, and Tanegashima is the northernmost island [1]. Although sugarcane is mostly used for refined sugar production [2,3], other sugarcane products are also important for the local economy, society, and industry. Sugarcane juice has been consumed as a beverage [4,5], and its health benefits have been highlighted [5]. Noncentrifugal sugar (NCS) has a more favorable effect on health than refined sugar because of its nutritional and functional value [5,6]. In Japan, a type of NCS called “Kokuto” [7] is a famous local specialty product of the Nansei Islands. Kokuto is not only eaten directly but also reprocessed as a substitute for refined sugar or ingredients for sweets and liquors; for instance, Japanese spirits made from Kokuto are called “Kokuto-shochu” [8]. Molasses is a byproduct of refined sugar production, and the effective utilization of its health function has attracted great attention [9]. Various products are derived from sugarcane, and the constituents of sugarcane directly influence their qualities.

The amino acids in sugarcane juice are relevant to the nutritional values of its processed products [7]. The concentration and composition of amino acids in sugarcane juice affect the texture, taste, and flavor of processed products [7]. The Maillard reaction forms various products, such as color pigments and volatile substance, which characterize NCS [7,10,11] and Kokuto-shochu [8]. NCS contains a processing contaminant called acrylamide [6,12], which is generated by the Maillard reaction of asparagine [13,14]. The acrylamide content of NCS is affected by the concentration of asparagine in sugarcane juice [15]. Therefore, the analysis of amino acid composition in sugarcane juice is important for improving and controlling the quality and safety of processed products made from sugarcane. Furthermore, in sugarcane juice, the content of amino acids is impacted by nitrogen fertilization [16,17,18]. “Amino-N” [19] was defined by Keating et al. as “nitrogen in amino acids” [19] and mirrors the nitrogen condition in the sugarcane [16,19]. Amino acid content may be used as a cue for estimating the appropriate amount of fertilization [16,18,19]. Therefore, amino acid evaluation in sugarcane juice could be an important approach to tackle the problem of soaring fertilizer prices and high environmental burden [16,18,19].

The goal of breeding sugarcane is to produce a cultivar whose phenotype meets the needs of farmers and sugar manufacturers. The characteristics include plants that yield more sugar or biomass, mature earlier, and resist damage by diseases and harmful insects, and labor-saving aspects, such as aptitude for machinery harvest (finer cane diameter) and multiratooning ability [1,20]. Little attention has been paid to constituents other than sugars (sucrose and reducing sugar) in sugarcane juice during sugarcane breeding programs in Japan. It is not enough to examine the relationships between the constituents and agronomic characteristics. Considering the importance of amino acid composition, it is expected that the utilization of the metabolic profile of sugarcane juice may help establish new breeding and cultivation methods.

A data-driven approach enables summarizing universal principles by analyzing vast data without a hypothesis. The data-driven approach has been applied in studies in the field of agricultural and food sciences, such as in yield estimation for the management of sugarcane cultivation [21] and the visualization of usual nutrient ingestion [22]. In this study, we used a data-driven approach to analyze agronomic data obtained from breeding programs every year. In Japanese sugarcane breeding programs, data handling and evaluation have not been systemized. In some cases, breeders must judge whether there are any clones remaining from a few survey items. Thus, the data-driven approach can analyze large volumes of agronomic data comprehensively regardless of years of experience in breeding, enabling breeding programs to identify clones with potential.

Most studies targeting sugarcane metabolites have tested various parts of the plant, including the leaves [23,24,25,26], buds [27], roots [23,26], rinds [28], and stalks [23,25,26,27,29]. Few studies have analyzed sugarcane juice collected from breeding programs at research institutes [30] or from sugar-producing factories [7,16,19]. Therefore, this study focused on sugarcane juice obtained from breeding programs for commercial cultivars. In the breeding program, plants were cultivated using common cultivation methods, and sugarcane juice was prepared using the same methods as a quality evaluation in factories. Practical knowledge could be obtained under conditions similar to those of farmers.

Therefore, this study aims to provide a method based on a data-driven approach for unambiguously interpreting agronomic data, regardless of the level of experience and knowledge of breeding and cultivation methods. Another aim of the study is to apply the established method to clarify the relationship between free amino acid contents in sugarcane juice and agronomic characteristics to improve breeding and cultivation.

## 2. Materials and Methods

### 2.1. Plant Materials and Cultivation Methods

Sugarcane was cultivated in an experimental field at Tanegashima Research Station, Kyushu-Okinawa Agricultural Research Center, NARO, Japan (30°43′ N, 131°04′ E), from 2018 to 2021, as a “Local Yield Evaluation” [1], “Regional Adaptability Test” [1], and “4th selection” [1] under the breeding program [1].

We used six cultivars: Harunoogi [1,31], KTn03-54 [32], Ni22 [1,33], NiTn18 [1,20,34], NiF8 [1,20], and NCo310 [1,20], as well as 21 unreleased clones under selection (KR05-619, KR11-655, KR12-154, KR12-251, KR12-316, KR13-130, KY10-1060, KY10T-531, KY11-1020, KY11-1199, KY11-1207, KY11-836, KY12-544, KY12-550, KY12-671, KY12-704, KY12-872, KY12-919, KY12T-5007, KY12T-5024, and KY12T-5050). Crossbreeding information for each sampled cultivar and clone is listed in Table S1.

The cropping types were new planting and ratoon (first ratoon and second ratoon). New plantings in Tanegashima are primarily planted in spring [2] because sugarcane planted in summer is influenced by winter low temperatures [1]. The first ratoon means sprouts from newly harvested canes, and the second ratoon means sprouts from first harvested canes. Three cropping types were used: new planting, first ratoon, and second ratoon; their planting (or previous harvesting) seasons were April or May–next January, January–November (early December), and November–next November, respectively [30].

The numbers of samples (experimental plots) by cultivars and clones, cropping type, and year are shown in Table S2.

We cultivated plants in accordance with customary agricultural practices [30,35], that is, single-bud stalks were embedded in the field with the plant density generated using a 110 cm width of furrow and 15 cm plant distance for new planting. Chemical fertilizer was applied as a basal dressing (7.2 g N m^−2^, 12.0 g P_2_O_5_ m^−2^, 6.0 g K_2_O m^−2^) and top dressing twice (4.5 g N m^−2^, 4.5 g K_2_O m^−2^) according to the regional cultivation guideline. The ridges were covered with the mulch film until intertillage and ridging in May. The soil in the experimental field was a type of volcanic soil, Silandic Andosol (Kuroboku). The experimental field was not irrigated during the cultivation period.

Experimental plots were designed for the local yield evaluation test (regional adaptability test) and fourth selection by triplicate and duplicate randomized block design, and each plot was 3.3 m^2^ (3.0 m long × 1.1 m wide furrow) and 2.2 m^2^ (2.0 m long × 1.1 m wide furrow) in size, respectively.

### 2.2. Agronomic Characteristics of Sugarcane

We investigated agronomic characteristics from the growing season to the postharvest of sugarcane, according to the sugarcane research standard [36,37,38]. The detailed definition of survey items is compiled in Table S3.

The growth surveys were conducted in spring (from the end of March to April), July, and October in each experimental plot. We determined Brix values of the upper, middle, and lower internodes of stalks using a handheld refractometer (MASTER-Pα, Atago Co., Ltd., Tokyo, Japan or SK-100R, Sato Keiryoki Mfg. Co., Ltd., Tokyo, Japan) in October.

For the postharvest survey in the local yield evaluation test, we manually harvested the entire experimental plot (3.3 m^2^ × 3), counted the number of stalks, and weighed them after removing all leaves and cane tops. We selected 10 or 6 millable stalks at random as representative samples of experimental plots and measured the weight of tops, the lengths and diameters of millable stalks, and the counted number of their internodes, and then calculated the averages.

### 2.3. Preparation of Sugarcane Juice and Bagasse

Stalks for which postharvest measurements were completed were used for juice extraction within 3 d after harvest. Sugarcane juice was prepared using the same method as in previous studies [30,39], which is as follows: The stalks were cut into two or three node lengths using a stem cutting machine (PC250-TM, Bunmei Noki K.K., Kagoshima, Japan). All stalks were shredded using a shredder (KS-MS, Matsuo Co., Ltd., Kagoshima, Japan). Then, we weighed 500 g of shredded stalks and compressed them by applying a pressure of 240 kgf/cm^2^ for ~1 min using a hot-press machine (CSS-NP-02H, Nittoku Kikai Kogyo K.K., Chiba, Japan). Approximately 12 mL of extracted sugarcane juice was sampled from a 15 mL conical tube and then frozen and stored at −30 °C until use. The remaining juice was used for quality evaluation. According to a common custom, wet bagasse was weighed and placed in cloth bags, which were weighed again after drying for ≥48 h in an industrial test chamber (EPRL-2700-2T, Isuzu Seisakusho K.K., Niigata, Japan) at 100 °C. We calculated fiber content from bagasse weight. A detailed explanation of the post-compression experiment is presented in Table S3.

### 2.4. Evaluation of Sugarcane Juice Quality

RI Brix values of sugarcane juice were measured using a digital refractometer (RX-5000, Atago Co., Ltd., Tokyo, Japan). Sugarcane juice was clarified using the methods based on a previous report [40] for international sugar scale analysis. In total, 100 mL of juice was mixed with ~3 g of calcium hydroxide (Guaranteed Reagent, Fujifilm Wako Pure Chemical Corporation, Osaka, Japan) and stirred using a magnetic stirrer. Approximately 7 g of aluminum (III) chloride hexahydrate (Guaranteed reagent for JIS, Kanto Chemical Co., Inc., Tokyo, Japan) was added to this mix and stirred. The solution was filtered through filter paper (No. 1, Toyo Roshi Kaisha, Ltd., Tokyo, Japan). The filtrates were measured using the international sugar scale with an automatic polarimeter (MCP200 Sucromat, Anton-Paar Japan K.K., Tokyo, Japan).

### 2.5. Quantitative Analysis of Free Amino Acids in Sugarcane Juice

Samples of frozen juice were sent from Tanegashima Research Station to Koshi Research Station, Kyushu-Okinawa Agricultural Research Center, NARO, Japan (32°88′ N, 130°74′ E) by frozen delivery and stored at −20 °C until use.

To prepare samples for amino acid analysis, frozen sugarcane juice was thawed at 4 °C in a low-temperature room, shaken, and dispensed to approximately 10 mL. Then, ~1 g of diatomaceous earth (Tokusen Super Light, Tokyo Konno Co., Ltd., Tokyo, Japan) was added as a filter aid, shaken to prepare a suspension, and filtered through filter paper (No. 2, Toyo Roshi Kaisha, Ltd., Tokyo, Japan) with pleats. Subsequently, 0.5 mL of the filtrate was transferred to an Amicon Ultra-0.5 filter unit (3K, Merck KGaA, Darmstadt, Germany) set in the filtrate collection tube. Centrifugal filtration was performed (4 °C, 14,000 rpm (20,800× *g*), 30 min). The filtrate was diluted with lithium citrate buffer (MCI Buffer PF-1, Mitsubishi Chemical Corporation, Tokyo, Japan) 10, 20, or 40 times and stored at −20 °C until analysis. The sample was thawed at 4 °C in a refrigerator and mixed well by vortexing before analysis.

The quantitative analysis of free amino acids was performed using High-speed Amino Acid Analyzer (L-8800, Hitachi High-Tech Science Corporation, Tokyo, Japan) according to the method in the manufacturer’s instructions. As the eluent, we used buffer for the High-speed Amino Acid Analyzer PF-SET (for physiological fluid analysis, Mitsubishi Chemical Corporation, Tokyo, Japan). Peak detection was performed with the post-column derivatization method (absorbance at 570 nm or 440 nm [L-proline]) using Ninhydrin Coloring Solution Kit (Fujifilm Wako Pure Chemical Corporation, Osaka, Japan).

Amino acid standard mixture (Type H, Fujifilm Wako Pure Chemical Corporation, Osaka, Japan) and amino acids not included in the standard mixture, such as γ-aminobutyric acid (GABA), L-glutamine, and L-asparagine (Fujifilm Wako Pure Chemical Corporation, Osaka, Japan), were used as standards. Data analysis was performed with the one-point calibration curve quantification method using the analyzer’s accessory software (L-8800 AAA System Manager, Hitachi High-Tech Science Corporation, Tokyo, Japan).

Twenty types of amino acids were detected. Based on the manufacturer’s manual, for the injection volume and dilution rate, all values < 0.04 μmol/mL were considered undetected. We excluded L-cysteine, L-methionine, and L-proline from analysis and quantified 17 amino acids: L-alanine, L-arginine, L-asparagine, L-aspartate, L-glutamate, L-glutamine, glycine, L-histidine, L-isoleucine, L-leucine, L-lysine, L-phenylalanine, L-serine, L-(-)-threonine, L-tyrosine, L-valine, and GABA.

### 2.6. Statistical Analysis

We used Excel (version 2308, Build 16731.20550), R (version 4.1.2 (2021-11-01)—“Bird Hippie”) [41], and Rstudio (version 2021.9.0.351) [42] for data management and analysis. Data processing was carried out using the R packages “reshape2” [43], “tidyverse” [44], and “readxl” [45].

Principal component analysis (PCA) was applied to the 12 agronomic characteristics. These were common survey items measured during all tests and were used as parameters, except for data with missing values, and standardized before analysis. Hierarchical cluster analysis (HCA) of 27 sample cultivars and clones was based on the Ward method and Euclidean distance. Sample data included standardized average values of 12 agronomic characteristics from all experimental plots during 2018–2020, with respect to each cultivar and clone. Table S2 shows the sample number in detail. PCA and HCA were performed on R.

*p* < 0.05 was considered statistically significant. Multiple comparisons were performed using the Tukey–Kramer test or the Steel–Dwass test with the R package “NSM3” [46], depending on the results of the Shapiro–Wilk normality test. Correlations were analyzed using the “psych” package [47].

The R packages “ggplot2” [48], “ggarchery” [49], “ggrepel” [50], “patchwork” [51], “dendextend” [52], and “ggsignif” [53] were used to generate the figures in this study. Part of the color palette is based on “RColorBrewer” [54].

## 3. Results

### 3.1. Data-Driven Approach to Evaluate the Agronomic Characteristics of Sugarcane

#### 3.1.1. Visualization of Agronomic Characteristics Using a Data-Driven Approach

**Figure 1:**
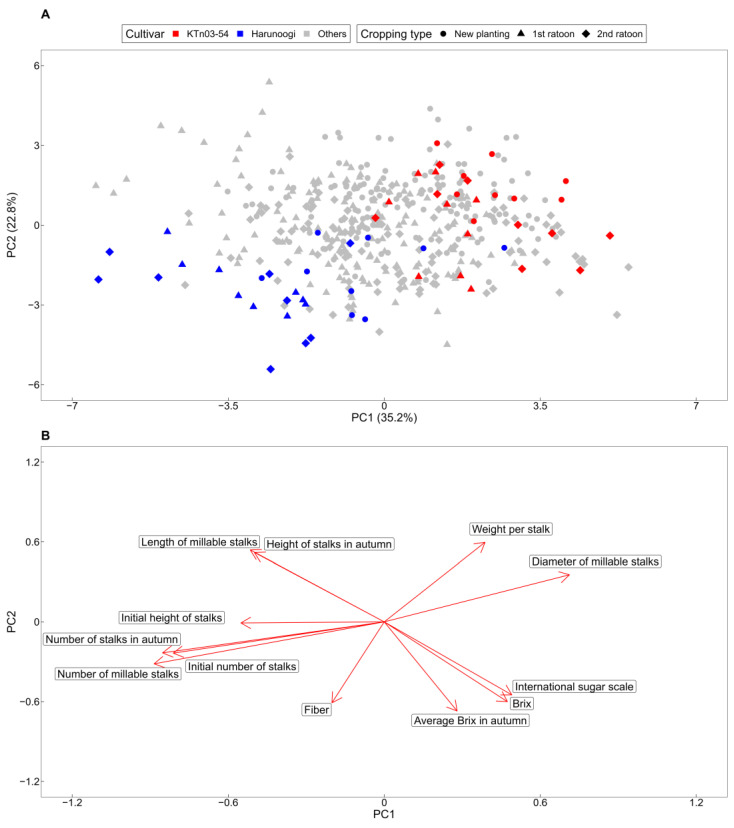
Principal component analysis (PCA) of data for the agronomic characteristics of sugarcane: (**A**) PCA score plot based on 12 agronomic characteristics of samples from 2018 to 2020 (*n* = 460); (**B**) loading plot based on the same analysis as the score plot A.

We visualized the agronomic characteristics of sugarcane cultivars and clones using a data-driven approach. PCA was performed to overview sample distribution and relationships of 12 agronomic characteristics measured regardless of cropping type or stage of selection (Figure 1). The contribution ratios of PC1 and PC2 were 35.2% and 22.8%, respectively. Except for some new planting samples, KTn03-54 and Harunoogi were clearly separated based on standard cultivars (Figure 1A). The loading plot (Figure 1B) showed that the vectors of number of millable stalks and diameter of millable stalks were opposite, contributing to KTn03-54 and Harunoogi, respectively.

**Figure 2:**
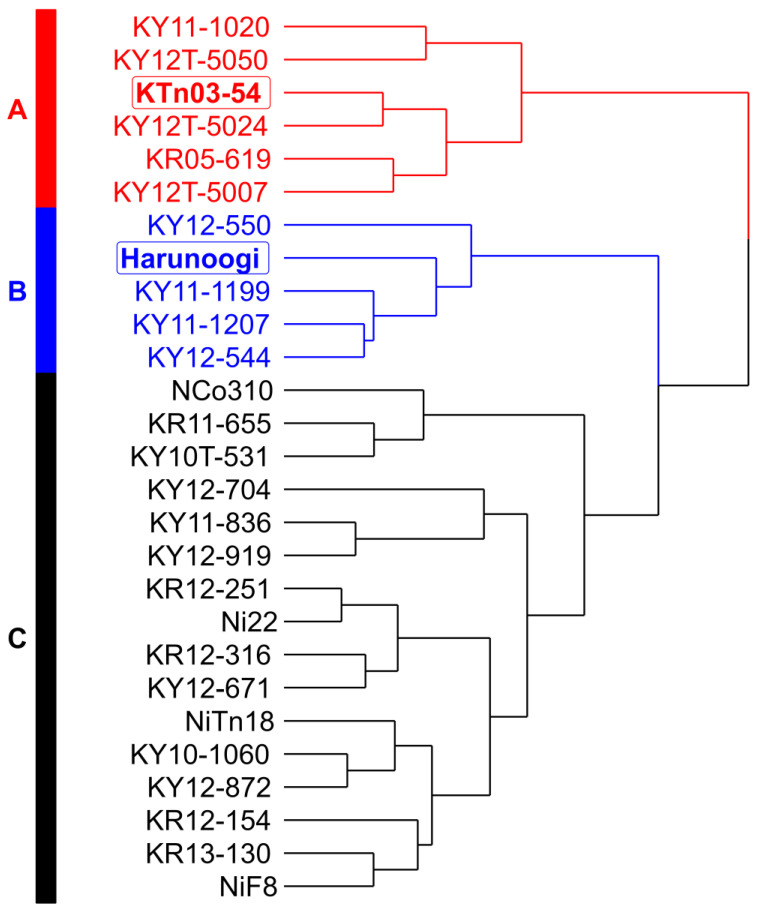
A dendrogram based on Hierarchical cluster analysis (HCA), calculated from 12 agronomic characteristics.

HCA (Figure 2) was calculated based on the average values of agronomic characteristics shown in Figure 1. Twenty-seven sugarcane cultivars and clones were divided into groups A–C by HCA. The results showed 3 groups. KTn03-54 and Harunoogi, which were distinctive cultivars, were included in groups A and B, respectively. Consistent with the results of PCA, groups A and B were separate. In addition, the other cultivars used in this study were categorized as group C.

#### 3.1.2. Analysis of the Agronomic Characteristics of the Three Groups of Sugarcane

**Figure 3:**
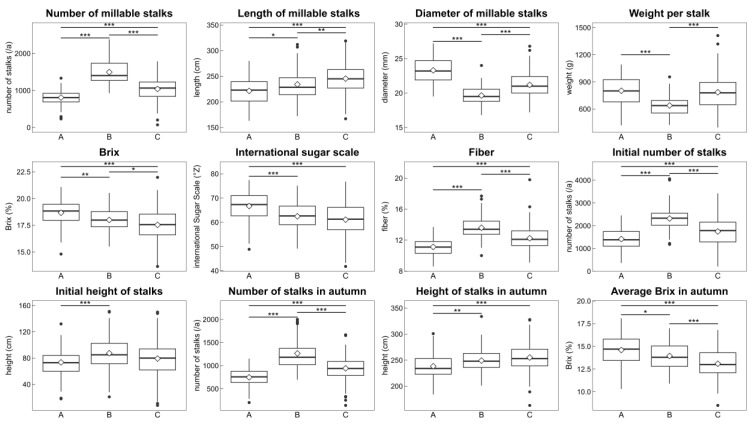
Comparison of 12 parameters between groups classified using HCA. We illustrate the parameters of agronomic characteristics used for HCA as box plots, arranged from left to right by groups A–C. Group A comprised 6 cultivars and clones, including KTn03-54; group B comprised 5 cultivars and clones, including Harunoogi; and group C comprised the remaining 16 cultivars and clones. Sample numbers of agronomic characteristics without length of millable stalks were *n* = 99, 75, and 290 for A, B, and C, respectively. Missing data for length of millable stalks are shown in Table S2. Diamonds indicate the means of each group. Statistical significance between groups was calculated using the Steel–Dwass test, except for the diameter of millable stalks, Brix, and average Brix in autumn. For these, the Tukey–Kramer test was used; *** *p* < 0.001, ** *p* < 0.01, * *p* < 0.05.

The data-driven approach showed 3 groups of sugarcane cultivars and clones based on the 12 agronomic characteristics. All these characteristics were visualized as box plots to confirm their contribution to this grouping, and statistically significant differences were calculated for them (Figure 3). Group A had the largest diameter of millable stalks, the heaviest weight per stalk, and the highest sugar content. Group B had the highest number of millable stalks and the highest fiber content. Group C had median values for the diameter of millable stalks, the number of millable stalks, weight per stalk, and the longest length of millable stalks and height of stalks in autumn. The sugar content of group C was the lowest.

### 3.2. Amino Acid Profiling of Sugarcane Juice

#### Features of Free Amino Acids among the Three Groups Have Different Agronomic Characteristics

**Figure 4:**
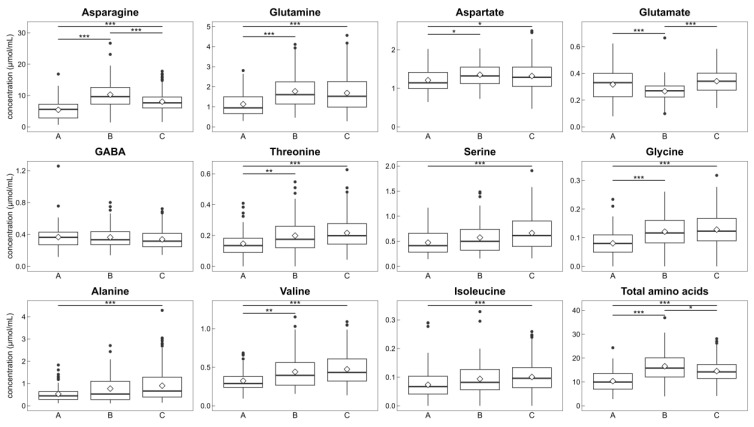
Comparison of amino acid content among the three groups. Eleven free amino acids and total amino acid concentrations in sugarcane juice are shown as box plots by groups, which were classified using HCA. Groups A, B, and C are arranged from left to right. Sample numbers of amino acids without asparagine, glutamate, and total amino acids are *n* = 99, 75, and 290 for A, B, and C, respectively. Missing data for asparagine, glutamate, and total amino acids are shown in Table S2. Diamonds indicate the means of each group. Statistical significance between groups was calculated using the Steel–Dwass test; *** *p* < 0.001, ** *p* < 0.01, * *p* < 0.05. Abbreviation: GABA, γ-aminobutyric acid.

We analyzed free amino acid concentrations in sugarcane juice to determine the relationship between metabolites and agronomic characteristics (Figure 4). Eleven free amino acids were abundant, and the total free amino acid concentration was calculated as the sum of the detected free amino acid contents. The content of several amino acids differed among groups with different agronomic characteristics. Group B exhibited the highest average concentrations of asparagine, glutamine, and aspartate with the lowest concentration of glutamate. The total free amino acids showed a similar trend as these amino acids. There was no significant difference in GABA concentration among the groups. Group A contained the lowest concentration of free amino acids, except GABA and glutamate in these 11 amino acids. Group C had the highest average concentration of some amino acids, including alanine, which had a relatively high concentration.

Moreover, sugar content and free amino acid content did not correlate highly, although threonine (−0.59, −0.64), serine (−0.54, −0.57), glycine (−0.52, −0.56), and alanine (−0.57, −0.60) were moderately correlated with Brix and international sugar scale, respectively (Table S4). No high correlation of amino acids with other agronomic characteristics was observed.

**Figure 5:**
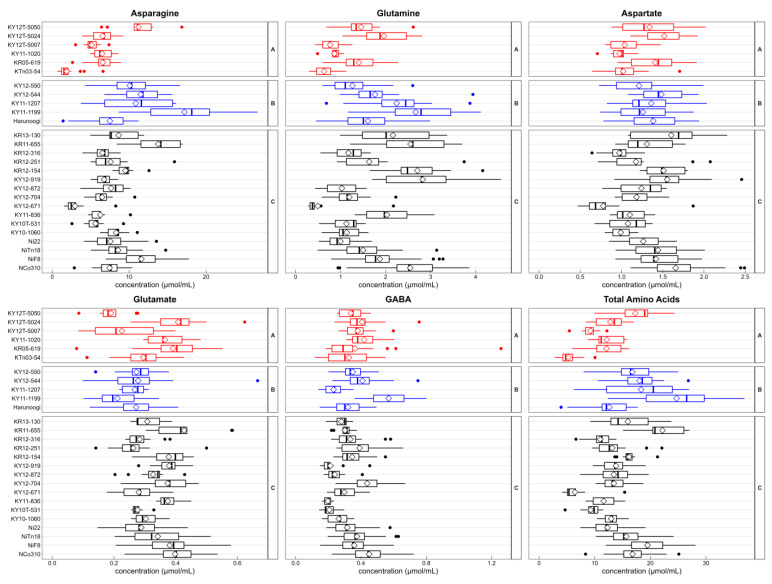
Comparison of amino acid content between cultivars and clones. Five free amino acids and total concentration in sugarcane juice are shown as box plots by groups classified using HCA, as well as by cultivars and clones. Groups A–C are arranged from top to bottom. The groups are represented by colors corresponding to the grouping in Figure 2. Sampled sugarcane were all cropping types, harvested from 2018 to 2021. Sample numbers differed depending on cultivars and clones, *n* = 5–39. The number of data for each cultivar or clone is shown in Table S2. Diamonds indicate the means of each cultivar or clone.

Groups A and B were more different in agronomic characteristics, except stalk length and sugar content, and group C had intermediate characteristics. Therefore, we focused on differences between groups A and B in the content of amino acids (Figure 5). Although free amino acid concentration varied in the same group depending on cultivars and clones, overall, tendencies were the same as those shown in Figure 4.

**Figure 6:**
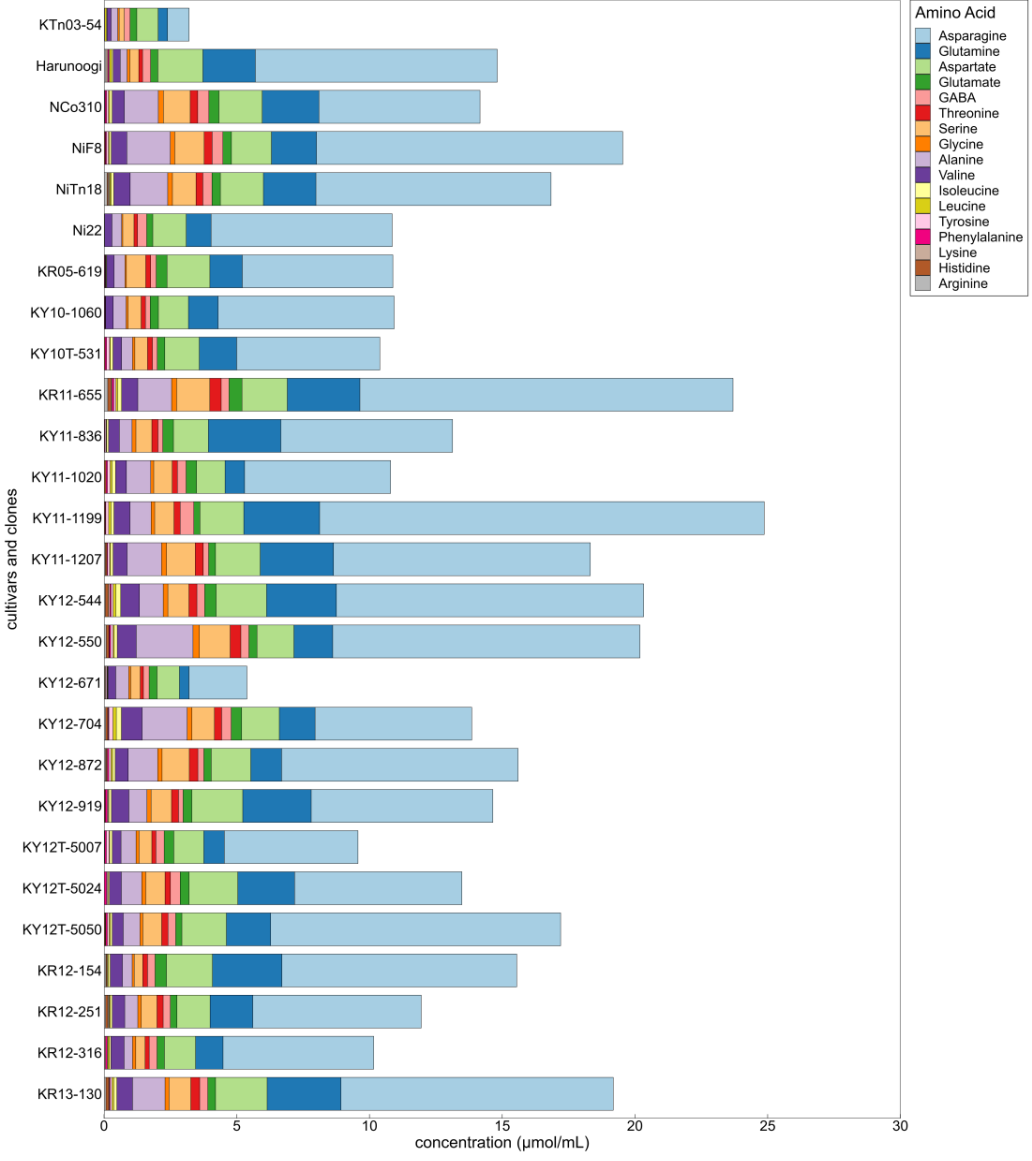
Differences in amino acid composition in sugarcane cultivars and clones. The stacked bar graph illustrates the average content of 17 amino acids in sugarcane juice from 6 cultivars and 21 clones. Sampled sugarcanes were newly planted and harvested in January 2019. *n* = 5; NiF8, Ni22, and NCo310 contained duplicate test plots at the fourth selection. *n* = 3; other cultivars and clones.

Amino acid profiles and compositions varied similarly to amino acid concentrations. There were some differences in the composition of amino acids when the total amino acid concentration was almost the same (Figure 6). Free amino acid concentrations and ratios in sugarcane juice were likely to present a unique characteristic depending on the cultivar and clone (Figure 5 and Figure 6).

## 4. Discussion

In this study, we used a data-driven approach to evaluate agronomic data from a sugarcane breeding program. Our findings showed that KTn03-54 and Harunoogi had contrasting agronomic characteristics (Figure 1A). According to cultivar registration reports [31,32], the weight per stalk of KTn03-54 was heavier than that of NiF8, and Harunoogi had a higher stalk number than NiF8. For >20 years, NiF8 has been the major cultivar in Kagoshima [1] and is cultivated as an example cultivar in breeding programs. This was consistent with our results (Figure 1), that is, the loading factors “weight per stalk/stalk diameter” and “number of millable stalks” contributed to KTn03-54 and Harunoogi, respectively, on PCA visualization. Because the yield of the estimated available sugar is calculated by commercial cane sugar and the weight of millable stalks, which is the product of weight per stalk and stalk number [55], weight per stalk and the number of millable stalks are keys for classifying yield type [55]. Among Japanese sugarcane breeders, the “stalk weight” [55] type and “stalk number” [55] type are common yield type classifications of cultivars [32,55]. Among the cultivars used in this study, NiF8 is more of a stalk weight type, and Ni22 represents a “stalk number type” [56] of cultivar. The yield type of a clone under selection was decided by comparison with standard cultivars, such as NiF8 and Ni22 [31,32]. According to Figure 2, KTn03-54 and Harunoogi belong to distant groups with different agronomic characteristics, as illustrated in Figure 3. They represent two different yield types more definitely than NiF8 and Ni22. The clones were also judged for cultivar registration by comprehensive evaluation based on breeder experiences in Japanese breeding programs. However, breeders have not always processed enormous data obtained from breeding programs objectively and unambiguously. In particular, at the early stage of the breeding program, there are too many clones for breeders to select all potential clones. Therefore, a data-driven approach is suitable for handling large numbers of clones and data. The method described in this study is potentially applicable to the classification of yield types. It should also help understand the trend of yield in different years or examine the agronomic characteristics of other clones under selection. This approach can facilitate the evaluation of sugarcane clones.

Our results shown in Figure 4 suggest that the metabolism of several amino acids differs among genotypes with different agronomic characteristics. Singh et al. [57] reported that in leaves, nitrate reductase, glutamate dehydrogenase, and glutamine synthetase of the more tillering and germinating cultivar Co.S.8118 were more active than those of the lower tillering cultivar Co.S.8315. Glutamine synthetase catalyzes the synthesis of glutamine from glutamate and ammonia; therefore, cultivars and clones with a higher stalk number can have lower glutamate concentrations (Figure 4). Ferreira et al. also revealed that glutamate and some amino acids in the stalk had a negative relationship with the sprouting rate [27]. Yuan et al. showed differences in alanine and asparagine contents in the stalk between two sugarcane cultivars with different rind colors and agronomic characteristics (FN41 and 165204) [25]. Perlo et al. found that glutamate at a ripe internode and alanine and isoleucine at an unripe internode were positively correlated with Brix in midterm [29], but alanine showed the opposite trend in our study. The correlation between agronomic characteristics and amino acids remains to be clarified and must be further studied.

Cultivars and clones have been proposed to be the factors influencing the concentration and composition of free amino acids in sugarcane juice [16,18,58,59]. This is consistent with our results (Figure 5 and Figure 6). Other factors affecting them were the growing period [16,30,58,59,60], segment of stalk [58], water condition [16,18,58], and nitrogen fertilization [16,17,18]. In this study, because the growing period and harvest time differed depending on the cropping type, the effect of each of these factors on amino acids was not identified. Nevertheless, the data-driven approach indicated that cultivars and clones dominantly affected amino acid metabolism compared with growing period, cropping type, and harvest year.

The concentration and composition of free amino acids varied (Figure 5 and Figure 6) compared with the sugar content because breeders have aimed to achieve higher sugar yields and have not focused on amino acid content in breeding programs. Therefore, there were various amino acid characteristics in clones. In other words, diverse amino acid compositions could coexist with a high sugar content. These findings should be useful for selecting potential clones for NCS production in breeding programs because the amino acid compositions affect the unique texture, flavor, and taste of the NCS by the Maillard reaction during the NCS manufacturing process [7]. In addition, control of the compositions, i.e., decreasing the ratio of asparagine, is also valuable for reducing the acrylamide content in the final products.

Asparagine was found to be the primary amino acid in sugarcane juice, positively correlating with the total amino acid content (Figure 3, Figure 4 and Figure 5), consistent with other studies [12,17,19,58,59,60]. The structure of asparagine is suitable for the efficient transport and storage of nitrogen because it contains two amino groups [61]. The tissue of stalk and xylem sap of sugarcane are mostly occupied by the organic nitrogen of asparagine [23,26], which in stalks is responsive to nitrogen fertilization [26], and it seems to be a form of transport and storage of nitrogen [19,23,26]. Regarding the influence of fertilization, content of “amino-N” [16] increased in sugarcane juice with increasing amounts of nitrogen fertilization [16]. Parish reported that the concentrations of asparagine, glutamine, and aspartate in sugarcane juice increased as nitrogen fertilization increased (0, 30, and 60 Nkg/acre) [17]. According to Chapman et al. [18], asparagine is more reactive to changes in nitrogen fertilization than the total amino acid content in “mixed juice” [18]. They also reported differences in response to fertilization among cultivars affect amino acid contents [18], which is consistent with the finding of other study [16].

Bassi et al. [24] discussed a cultivar exhibited less sensitivity to a high-nitrogen fertilization in which nitrogen use efficiency was comparatively low in high-nitrogen fertilization. This cultivar contained more asparagine, glutamine, and glutamate in the part from the base (nearly dewlap) to the middle of leaves under the high-nitrogen condition [24]. They speculated that the ammonium anabolism of glutamine or senescence of the leaves is vigorous in this cultivar [24]. Considering these reports [16,18,19,26] and the results of this study, we hypothesize that variations in free amino acids among cultivars and clones mirror the surplus and shortage of the required amount of nitrogen, depending on the fertilizer supplied to each cultivar and clone. Keating et al. concluded that it was useful to examine content of “amino-N” [16] to control the provision of nitrogen [16]. Measuring the concentration of amino acids is useful not only for controlling the quality of sugarcane products but also for assessing the characteristics of nitrogen metabolism in sugarcane cultivars and clones. Keating et al. [19] suggested the possibility that sugar-producing factories can analyze nitrogen from amino acids using near-infrared analysis with sugar content for quality-based transactions and provide feedback to farmers. This analysis method is easier and will help with the social implementation of findings on amino acid characteristics in sugarcane.

One of the significant problems in agriculture is the increase in production cost, increase in fertilizer price, and the need for sustainable agriculture by decreasing the environmental burden [16,18,19,26]. Therefore, it is essential to determine the standard amount of nitrogen fertilizer. However, standards for nitrogen fertilization based on cultivars are not well established in Japan. This is because it is difficult to calculate the amount of nitrogen fertilizer needed for each cultivar and every farm based on the nitrogen balance in each field from soil diagnosis [19]. According to this study, differences in nitrogen demand depending on cultivars and clones caused significant differences in free amino acid contents under the same amount of nitrogen fertilization. It will be possible to understand the characteristics of nitrogen metabolism in cultivars and clones based on the content of a specific free amino acid in the juice, which is easily obtained from samples during routine breeding. It is useful for breeders and farmers to assess the amount of nitrogen fertilization needed by cultivars and clones. The relationship between the amount of nitrogen fertilization needed and the free amino acid content will be discussed in future studies.

## 5. Conclusions

This study examined a data-driven approach applied to agronomic data from sugarcane breeding selection and clarified a correlation between them and amino acid profiles in sugarcane juice. The data-driven approach revealed the diversity and variability of each sample and divided 27 cultivars and clones into three groups based on 12 agronomic characteristics. Each group had characteristic features such as the largest diameter of millable stalks in group A and the highest number of millable stalks in group B. Significant differences in amino acids asparagine and glutamine were observed upon comparing free amino acid constituents between these groups. The concentration and composition of free amino acids varied when compared with sugar content. Moreover, cultivars and clones dominantly affected amino acid metabolism in sugarcane. Differences in nitrogen demand depending on cultivars and clones may be the cause of significant differences in free amino acid contents among cultivars and clones using the same nitrogen fertilization amount. The relationship between the amount of nitrogen fertilization needed and the free amino acid contents will be discussed in future studies. Our approach, which deals with a massive volume of data on agronomic characteristics, will be useful in assessing and evaluating the characteristics of potential clones under selection and accelerating breeding programs.

## Data Availability

The data presented in this article are available on request from the corresponding author due to intellectual property protection.

## Supplementary Materials

The following supporting information can be downloaded at: https://www.mdpi.com/article/10.3390/metabo14040243/s1, Table S1: Crossbreeding information about sampled cultivars and clones; Table S2: Survey items and definitions of agronomic characteristics in the sugarcane breeding program; Table S3: Number of samples for each cultivar and clone, cropping types by year; Table S4: Correlation coefficients between agronomic characteristics and free amino acid contents.
