# Supplementary material for: A Data-Driven Approach to Sugarcane Breeding Programs with Agronomic Characteristics and Amino Acid Constituent Profiling
Source: Metabolites. 2024 Apr 21;14(4):243. doi: 10.3390/metabo14040243 (PMC11052186; doi:10.3390/metabo14040243)
Supplement: Supplementary file 1 [file metabolites-14-00243-s001.zip › SupplementaryTable_FinalVersion240420.pptx]

## Slide 1
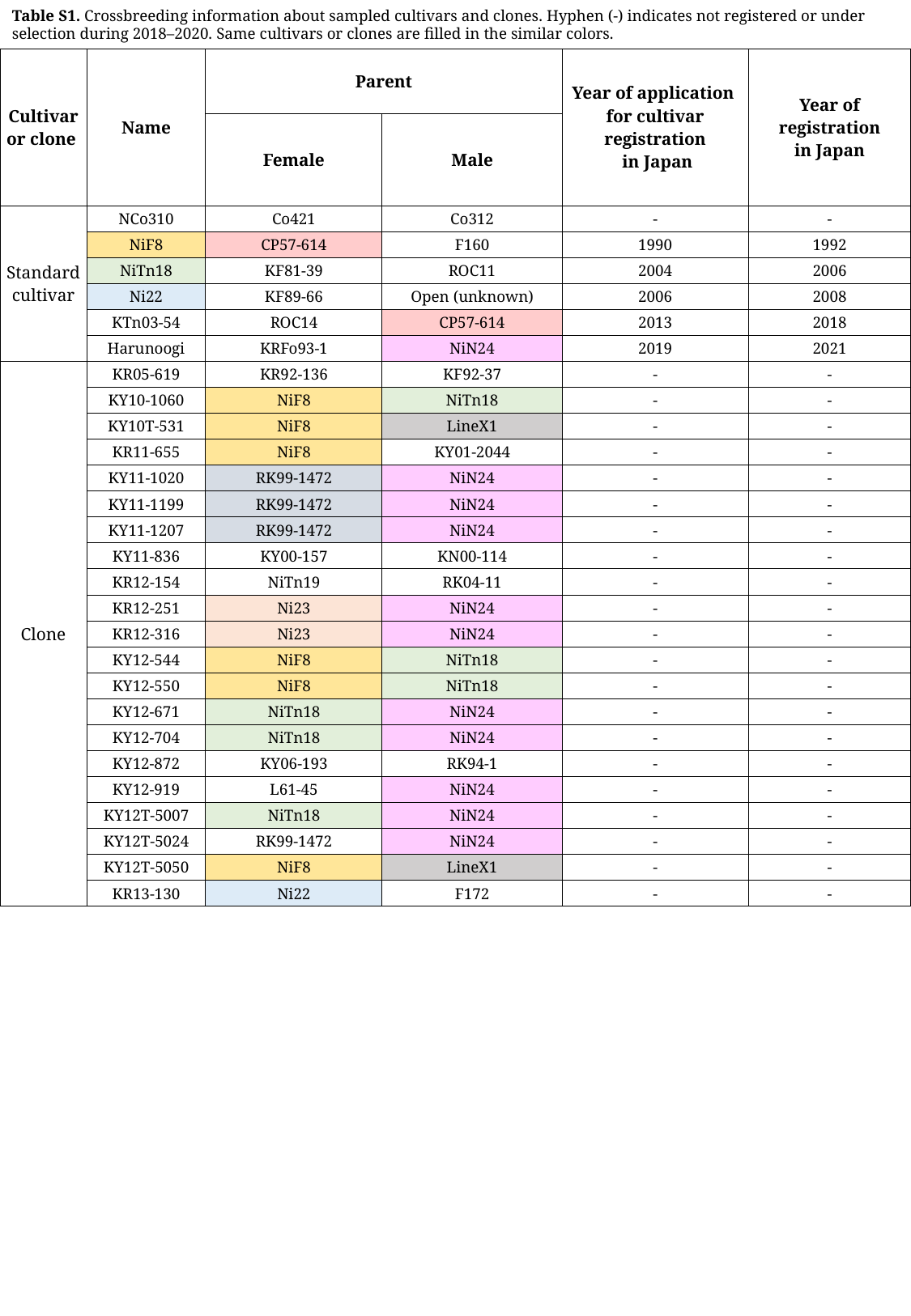

Table S1. Crossbreeding information about sampled cultivars and clones. Hyphen (-) indicates not registered or under selection during 2018–2020. Same cultivars or clones are filled in the similar colors.
| Cultivar or clone | Name | Parent | | Year of application for cultivar registration in Japan | Year of registration in Japan |
| --- | --- | --- | --- | --- | --- |
| | | Female | Male | | |
| Standard cultivar | NCo310 | Co421 | Co312 | - | - |
| | NiF8 | CP57-614 | F160 | 1990 | 1992 |
| | NiTn18 | KF81-39 | ROC11 | 2004 | 2006 |
| | Ni22 | KF89-66 | Open (unknown) | 2006 | 2008 |
| | KTn03-54 | ROC14 | CP57-614 | 2013 | 2018 |
| | Harunoogi | KRFo93-1 | NiN24 | 2019 | 2021 |
| Clone | KR05-619 | KR92-136 | KF92-37 | - | - |
| | KY10-1060 | NiF8 | NiTn18 | - | - |
| | KY10T-531 | NiF8 | LineX1 | - | - |
| | KR11-655 | NiF8 | KY01-2044 | - | - |
| | KY11-1020 | RK99-1472 | NiN24 | - | - |
| | KY11-1199 | RK99-1472 | NiN24 | - | - |
| | KY11-1207 | RK99-1472 | NiN24 | - | - |
| | KY11-836 | KY00-157 | KN00-114 | - | - |
| | KR12-154 | NiTn19 | RK04-11 | - | - |
| | KR12-251 | Ni23 | NiN24 | - | - |
| | KR12-316 | Ni23 | NiN24 | - | - |
| | KY12-544 | NiF8 | NiTn18 | - | - |
| | KY12-550 | NiF8 | NiTn18 | - | - |
| | KY12-671 | NiTn18 | NiN24 | - | - |
| | KY12-704 | NiTn18 | NiN24 | - | - |
| | KY12-872 | KY06-193 | RK94-1 | - | - |
| | KY12-919 | L61-45 | NiN24 | - | - |
| | KY12T-5007 | NiTn18 | NiN24 | - | - |
| | KY12T-5024 | RK99-1472 | NiN24 | - | - |
| | KY12T-5050 | NiF8 | LineX1 | - | - |
| | KR13-130 | Ni22 | F172 | - | - |

## Slide 2
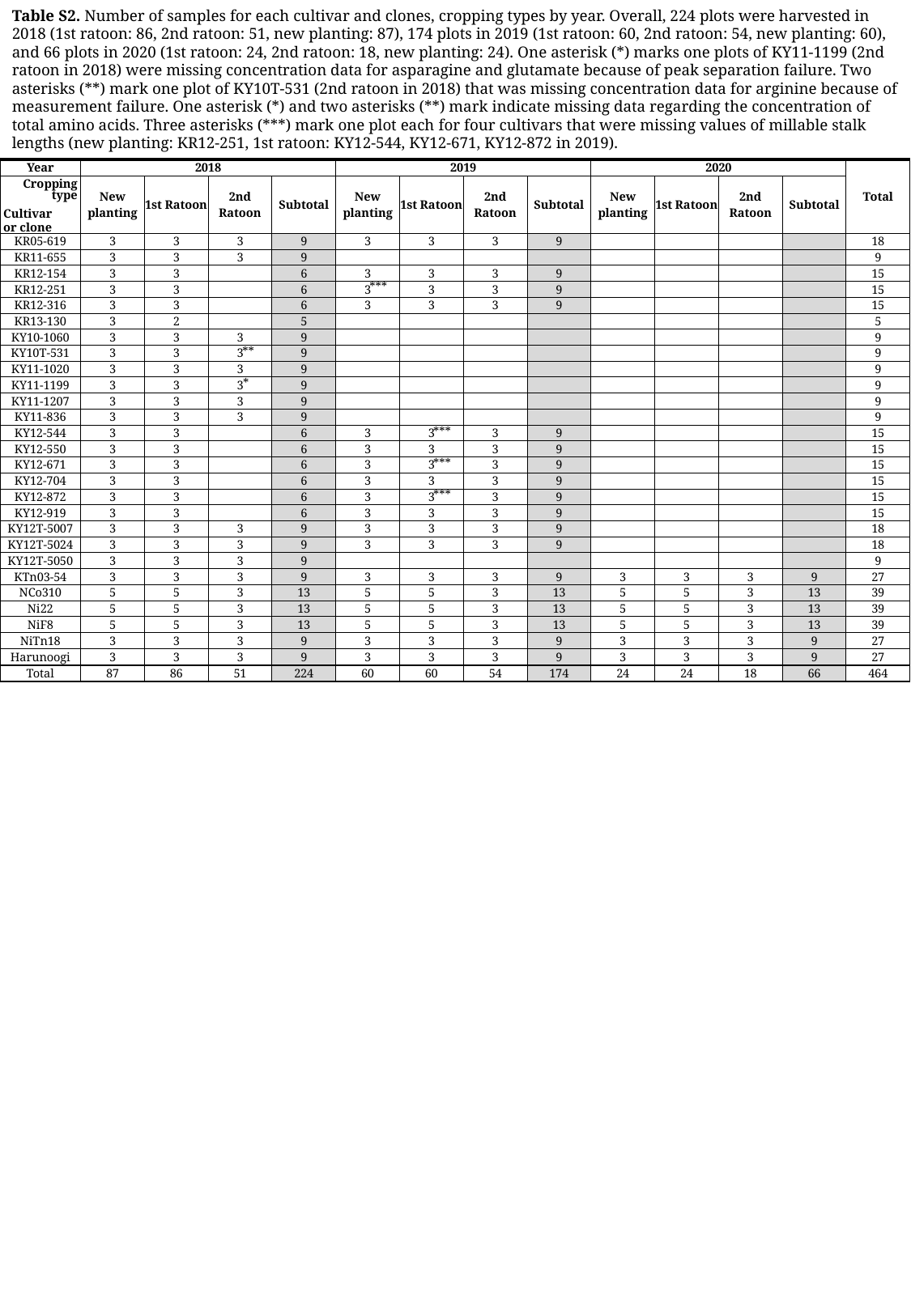

Table S2. Number of samples for each cultivar and clones, cropping types by year. Overall, 224 plots were harvested in 2018 (1st ratoon: 86, 2nd ratoon: 51, new planting: 87), 174 plots in 2019 (1st ratoon: 60, 2nd ratoon: 54, new planting: 60), and 66 plots in 2020 (1st ratoon: 24, 2nd ratoon: 18, new planting: 24). One asterisk (*) marks one plots of KY11-1199 (2nd ratoon in 2018) were missing concentration data for asparagine and glutamate because of peak separation failure. Two asterisks (**) mark one plot of KY10T-531 (2nd ratoon in 2018) that was missing concentration data for arginine because of measurement failure. One asterisk (*) and two asterisks (**) mark indicate missing data regarding the concentration of total amino acids. Three asterisks (***) mark one plot each for four cultivars that were missing values of millable stalk lengths (new planting: KR12-251, 1st ratoon: KY12-544, KY12-671, KY12-872 in 2019).
| Year | 2018 | | | | 2019 | | | | 2020 | | | | Total |
| --- | --- | --- | --- | --- | --- | --- | --- | --- | --- | --- | --- | --- | --- |
| | New planting | 1st Ratoon | 2nd Ratoon | Subtotal | New planting | 1st Ratoon | 2nd Ratoon | Subtotal | New planting | 1st Ratoon | 2nd Ratoon | Subtotal | |
| KR05-619 | 3 | 3 | 3 | 9 | 3 | 3 | 3 | 9 | | | | | 18 |
| KR11-655 | 3 | 3 | 3 | 9 | | | | | | | | | 9 |
| KR12-154 | 3 | 3 | | 6 | 3 | 3 | 3 | 9 | | | | | 15 |
| KR12-251 | 3 | 3 | | 6 | 3 | 3 | 3 | 9 | | | | | 15 |
| KR12-316 | 3 | 3 | | 6 | 3 | 3 | 3 | 9 | | | | | 15 |
| KR13-130 | 3 | 2 | | 5 | | | | | | | | | 5 |
| KY10-1060 | 3 | 3 | 3 | 9 | | | | | | | | | 9 |
| KY10T-531 | 3 | 3 | 3 | 9 | | | | | | | | | 9 |
| KY11-1020 | 3 | 3 | 3 | 9 | | | | | | | | | 9 |
| KY11-1199 | 3 | 3 | 3 | 9 | | | | | | | | | 9 |
| KY11-1207 | 3 | 3 | 3 | 9 | | | | | | | | | 9 |
| KY11-836 | 3 | 3 | 3 | 9 | | | | | | | | | 9 |
| KY12-544 | 3 | 3 | | 6 | 3 | 3 | 3 | 9 | | | | | 15 |
| KY12-550 | 3 | 3 | | 6 | 3 | 3 | 3 | 9 | | | | | 15 |
| KY12-671 | 3 | 3 | | 6 | 3 | 3 | 3 | 9 | | | | | 15 |
| KY12-704 | 3 | 3 | | 6 | 3 | 3 | 3 | 9 | | | | | 15 |
| KY12-872 | 3 | 3 | | 6 | 3 | 3 | 3 | 9 | | | | | 15 |
| KY12-919 | 3 | 3 | | 6 | 3 | 3 | 3 | 9 | | | | | 15 |
| KY12T-5007 | 3 | 3 | 3 | 9 | 3 | 3 | 3 | 9 | | | | | 18 |
| KY12T-5024 | 3 | 3 | 3 | 9 | 3 | 3 | 3 | 9 | | | | | 18 |
| KY12T-5050 | 3 | 3 | 3 | 9 | | | | | | | | | 9 |
| KTn03-54 | 3 | 3 | 3 | 9 | 3 | 3 | 3 | 9 | 3 | 3 | 3 | 9 | 27 |
| NCo310 | 5 | 5 | 3 | 13 | 5 | 5 | 3 | 13 | 5 | 5 | 3 | 13 | 39 |
| Ni22 | 5 | 5 | 3 | 13 | 5 | 5 | 3 | 13 | 5 | 5 | 3 | 13 | 39 |
| NiF8 | 5 | 5 | 3 | 13 | 5 | 5 | 3 | 13 | 5 | 5 | 3 | 13 | 39 |
| NiTn18 | 3 | 3 | 3 | 9 | 3 | 3 | 3 | 9 | 3 | 3 | 3 | 9 | 27 |
| Harunoogi | 3 | 3 | 3 | 9 | 3 | 3 | 3 | 9 | 3 | 3 | 3 | 9 | 27 |
| Total | 87 | 86 | 51 | 224 | 60 | 60 | 54 | 174 | 24 | 24 | 18 | 66 | 464 |
Cropping
type
Cultivar
or clone
***
**
*
***
***
***

## Slide 3
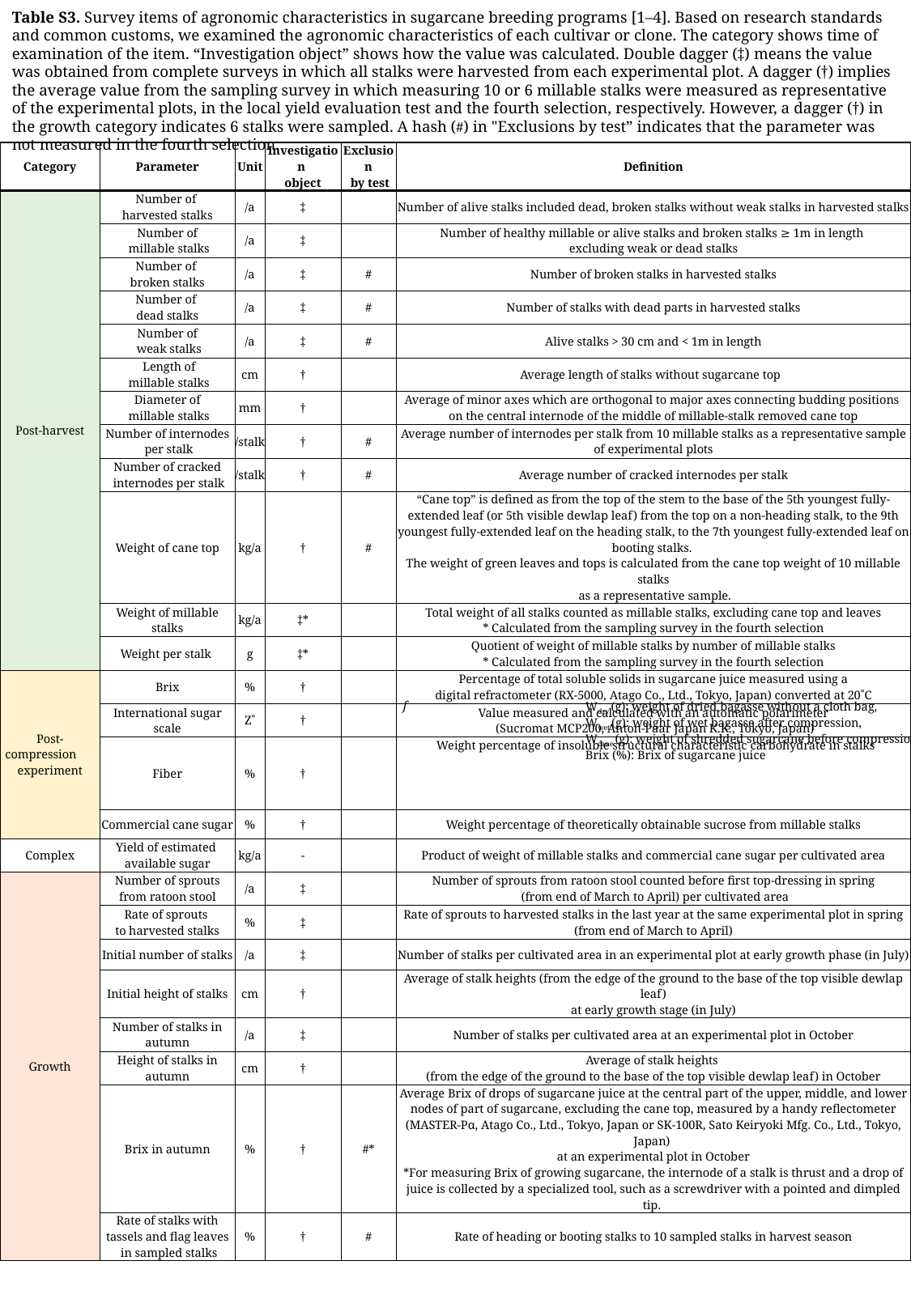

Table S3. Survey items of agronomic characteristics in sugarcane breeding programs [1–4]. Based on research standards and common customs, we examined the agronomic characteristics of each cultivar or clone. The category shows time of examination of the item. “Investigation object” shows how the value was calculated. Double dagger (‡) means the value was obtained from complete surveys in which all stalks were harvested from each experimental plot. A dagger (†) implies the average value from the sampling survey in which measuring 10 or 6 millable stalks were measured as representative of the experimental plots, in the local yield evaluation test and the fourth selection, respectively. However, a dagger (†) in the growth category indicates 6 stalks were sampled. A hash (#) in "Exclusions by test” indicates that the parameter was not measured in the fourth selection.
| Category | Parameter | Unit | Investigation object | Exclusion by test | Definition |
| --- | --- | --- | --- | --- | --- |
| Post-harvest | Number of harvested stalks | /a | ‡ | | Number of alive stalks included dead, broken stalks without weak stalks in harvested stalks |
| | Number of millable stalks | /a | ‡ | | Number of healthy millable or alive stalks and broken stalks ≥ 1m in length excluding weak or dead stalks |
| | Number of broken stalks | /a | ‡ | # | Number of broken stalks in harvested stalks |
| | Number of dead stalks | /a | ‡ | # | Number of stalks with dead parts in harvested stalks |
| | Number of weak stalks | /a | ‡ | # | Alive stalks > 30 cm and < 1m in length |
| | Length of millable stalks | cm | † | | Average length of stalks without sugarcane top |
| | Diameter of millable stalks | mm | † | | Average of minor axes which are orthogonal to major axes connecting budding positions on the central internode of the middle of millable-stalk removed cane top |
| | Number of internodes per stalk | /stalk | † | # | Average number of internodes per stalk from 10 millable stalks as a representative sample of experimental plots |
| | Number of cracked internodes per stalk | /stalk | † | # | Average number of cracked internodes per stalk |
| | Weight of cane top | kg/a | † | # | “Cane top” is defined as from the top of the stem to the base of the 5th youngest fully-extended leaf (or 5th visible dewlap leaf) from the top on a non-heading stalk, to the 9th youngest fully-extended leaf on the heading stalk, to the 7th youngest fully-extended leaf on booting stalks. The weight of green leaves and tops is calculated from the cane top weight of 10 millable stalks as a representative sample. |
| | Weight of millable stalks | kg/a | ‡\* | | Total weight of all stalks counted as millable stalks, excluding cane top and leaves \* Calculated from the sampling survey in the fourth selection |
| | Weight per stalk | g | ‡\* | | Quotient of weight of millable stalks by number of millable stalks \* Calculated from the sampling survey in the fourth selection |
| Post-compression　experiment | Brix | % | † | | Percentage of total soluble solids in sugarcane juice measured using a digital refractometer (RX-5000, Atago Co., Ltd., Tokyo, Japan) converted at 20˚C |
| | International sugar scale | Z˚ | † | | Value measured and calculated with an automatic polarimeter (Sucromat MCP200, Anton-Paar Japan K.K., Tokyo, Japan) |
| | Fiber | % | † | | Weight percentage of insoluble structural characteristic carbohydrate in stalks |
| | Commercial cane sugar | % | † | | Weight percentage of theoretically obtainable sucrose from millable stalks |
| Complex | Yield of estimated available sugar | kg/a | - | | Product of weight of millable stalks and commercial cane sugar per cultivated area |
| Growth | Number of sprouts from ratoon stool | /a | ‡ | | Number of sprouts from ratoon stool counted before first top-dressing in spring (from end of March to April) per cultivated area |
| | Rate of sprouts to harvested stalks | % | ‡ | | Rate of sprouts to harvested stalks in the last year at the same experimental plot in spring (from end of March to April) |
| | Initial number of stalks | /a | ‡ | | Number of stalks per cultivated area in an experimental plot at early growth phase (in July) |
| | Initial height of stalks | cm | † | | Average of stalk heights (from the edge of the ground to the base of the top visible dewlap leaf) at early growth stage (in July) |
| | Number of stalks in autumn | /a | ‡ | | Number of stalks per cultivated area at an experimental plot in October |
| | Height of stalks in autumn | cm | † | | Average of stalk heights (from the edge of the ground to the base of the top visible dewlap leaf) in October |
| | Brix in autumn | % | † | #\* | Average Brix of drops of sugarcane juice at the central part of the upper, middle, and lower nodes of part of sugarcane, excluding the cane top, measured by a handy reflectometer (MASTER-Pα, Atago Co., Ltd., Tokyo, Japan or SK-100R, Sato Keiryoki Mfg. Co., Ltd., Tokyo, Japan) at an experimental plot in October \*For measuring Brix of growing sugarcane, the internode of a stalk is thrust and a drop of juice is collected by a specialized tool, such as a screwdriver with a pointed and dimpled tip. |
| | Rate of stalks with tassels and flag leaves in sampled stalks | % | † | # | Rate of heading or booting stalks to 10 sampled stalks in harvest season |
Wdry (g): weight of dried bagasse without a cloth bag,
Wwet (g): weight of wet bagasse after compression,
Wcane (g): weight of shredded sugarcane before compression,
Brix (%): Brix of sugarcane juice

## Slide 4
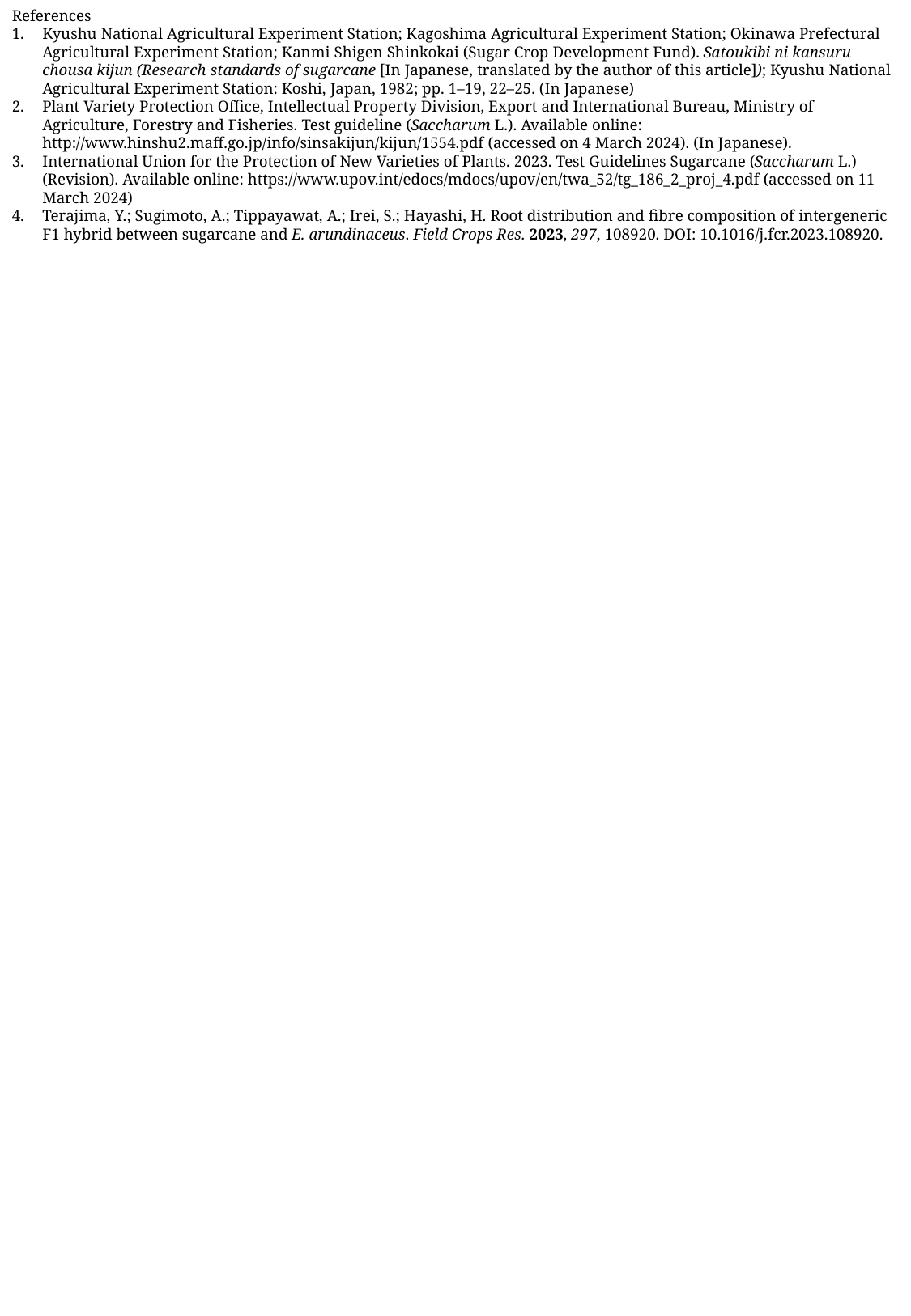

References
Kyushu National Agricultural Experiment Station; Kagoshima Agricultural Experiment Station; Okinawa Prefectural Agricultural Experiment Station; Kanmi Shigen Shinkokai (Sugar Crop Development Fund). Satoukibi ni kansuru chousa kijun (Research standards of sugarcane [In Japanese, translated by the author of this article]); Kyushu National Agricultural Experiment Station: Koshi, Japan, 1982; pp. 1–19, 22–25. (In Japanese)
Plant Variety Protection Office, Intellectual Property Division, Export and International Bureau, Ministry of Agriculture, Forestry and Fisheries. Test guideline (Saccharum L.). Available online: http://www.hinshu2.maff.go.jp/info/sinsakijun/kijun/1554.pdf (accessed on 4 March 2024). (In Japanese).
International Union for the Protection of New Varieties of Plants. 2023. Test Guidelines Sugarcane (Saccharum L.) (Revision). Available online: https://www.upov.int/edocs/mdocs/upov/en/twa_52/tg_186_2_proj_4.pdf (accessed on 11 March 2024)
Terajima, Y.; Sugimoto, A.; Tippayawat, A.; Irei, S.; Hayashi, H. Root distribution and fibre composition of intergeneric F1 hybrid between sugarcane and E. arundinaceus. Field Crops Res. 2023, 297, 108920. DOI: 10.1016/j.fcr.2023.108920.
